# Four Cases of Proximal Release-Type Colon Stents for Obstructive Rectal Cancer

**DOI:** 10.7759/cureus.59362

**Published:** 2024-04-30

**Authors:** Marina Jimba, Toshiyuki Enomoto, Yoshihisa Saida

**Affiliations:** 1 Department of Surgery, Toho University Ohashi Medical Center, Tokyo, JPN

**Keywords:** rectal cancer, obstruction, sems, self-expandable metallic colonic stent, proximal release, malignant colorectal obstruction, proximal release-type stent, case presentation

## Abstract

Malignant colonic obstruction can cause necrosis, bacterial translocation, electrolytic imbalance, and death; therefore, immediate decompression should be performed. Self-expandable metallic colonic stents are an established treatment for the decompression of malignant colonic obstructions. The use of stents that open from the distal side, which have been commonly used until now, requires caution because placing a stent on the dentate line can cause severe pain, and there is a possibility of cutting the stent during rectal resection of the distal side of the tumor. Therefore, we designed a new proximal-release-type colorectal stent for use in our hospital; it is 22 mm in diameter and 70 mm in length, which was placed using the over-the-wire method with a 16 Fr delivery system. We have encountered four cases in which it was appropriate as a bridge to surgical treatment. None of the patients experienced complications, such as bleeding, pain, or other incidents, after stent placement. Additionally, the stents were not affected by the surgical dissection of the rectum on the anorectal side of the tumor. Herein, we presented the four aforementioned cases and discussed the stenting techniques.

## Introduction

Colorectal cancer is one of the most common cancers worldwide [1], and approximately 10-20% of affected individuals present with colorectal obstruction [2-5]. Malignant colonic obstruction can cause necrosis, bacterial translocation, electrolytic imbalance, and death; therefore, decompression should be performed immediately after the discovery of the obstruction.

Owing to the potential for contamination, surgery for an obstructed colon has a higher-than-usual rate of surgical complications and mortality [6]. As one-stage anastomosis is associated with a high risk of dehiscence and other postoperative complications, many patients require colostomy, which adversely affects their quality of life (QOL). Therefore, the ideal approach involves conservative treatment to resolve bowel obstruction with the aim of avoiding emergency surgery, followed by elective surgery once the patient’s general condition has improved. However, decompression of the large intestine may be impeded by the ileocecal valve, which prevents reflux when nasogastric or nasointestinal ileus tubes are used. Therefore, colonic stents have been used as a bridge to surgery (BTS) to address obstructions since the 1990s; preoperative decompression occurs through the anus and acts as a bridge to elective surgery [7]. Currently, the transanal approach for preoperative decompression involves colonic stents [7] or transanal drainage tubes [8].

Self-expandable metallic stents (SEMS) are widely used to treat malignant colonic obstructions [9] and are indicated for placement in cases of malignant colorectal stenosis. Stents are intended to be temporary; however, they are also used to provide palliative treatment or as a bridge to surgery.

Unfortunately, placement of a stent at the site of an obstructed rectal lesion is difficult because of the high likelihood of pain and distress when the stent margin approaches the dentate line of the anal canal. It has also been reported that pain or discomfort is likely to occur when a stent is placed within 5 cm of the anal verge or the dentate line [10-13]. Additionally, the most commonly used stents open from the distal side, making it difficult to adjust their positioning on the anorectal side of the tumor; therefore, it is possible to cut the stent during any rectal resection performed distal to the tumor. To address this issue, we designed a new proximal-release-type colorectal stent for use in our hospital in June 2019. Herein, we reported four cases of BTS using a proximal release-type stent.

## Case presentation

We placed proximal release-type stents as a BTS in four patients with malignant colorectal obstruction at our hospital between October 2019 and December 2023 (Table 1).

**Table 1:** The four bridge-to-surgery cases The four bridge-to-surgery cases – outcomes of the newly developed proximal release-type self-expandable metallic stent placements

Case number	Age	Sex	Stricture length (mm)	Distance from the dentate line (mm)	Time from stent insertion to surgery (days)	CROSS before stent insertion	CROSS aftrer stent insertion	Stenting time (min)	Complications during stenting	Complications from stenting to surgery	Operation time (min)	Blood loss (ml)	Distal margin of the resected specimen (mm)	Postoperative complication	Postoperative hospital stay (days)
1	69	man	20	110	28	3	4	15	None	None	219	50	30	None	7
2	84	woman	35	120	24	1	4	15	None	None	153	0	20	None	9
3	55	man	50	100	18	2	3	12	None	None	262	90	40	None	10
4	79	woman	50	90	13	0	4	25	None	None	180	0	25	Minor leak	14

The median colorectal obstruction score (CROSS; Table 2) before stent placement was 1.5 (range: 0-3), which increased to 4 (range: 3-4) after stent placement, indicating an improvement in the condition of all four patients. The median procedure time was 15 min (range: 12-25 min), and there were no complications during stent insertion (bleeding, perforation, etc.) or during the interval between stent placement and surgery (migration, re-obstruction, pain, etc.).

**Table 2:** The ColoRectal Obstruction Scoring System The ColoRectal Obstruction Scoring System of the Japan Colonic Safe Procedure Research Group is available to the public on its home page [14].

Level of Oral Intake	Score
Requiring continuous decompressive procedure	0
No oral intake	1
Liquid or enteral nutrient	2
Soft solids, low-residue, and full diet with symptoms of stricture	3
Soft solids, low-residue, and full diet without symptoms of stricture	4

All patients underwent laparoscopic low anterior resection. The median operative time was 199.5 (range: 153-262) min, and the median blood loss was 25 (range: 0-90) mL. No intraoperative complications or conversion to open surgery were observed. There was one incidental postoperative complication: a minor anastomotic leakage requiring a two-week course of antimicrobial therapy prior to discharge. No other adverse events were observed. The median postoperative hospital stay was 9.5 (range: 7-14) days, and all patients had negative pathological margins.

Case 1 is presented as a representative.

**Figure 1:**
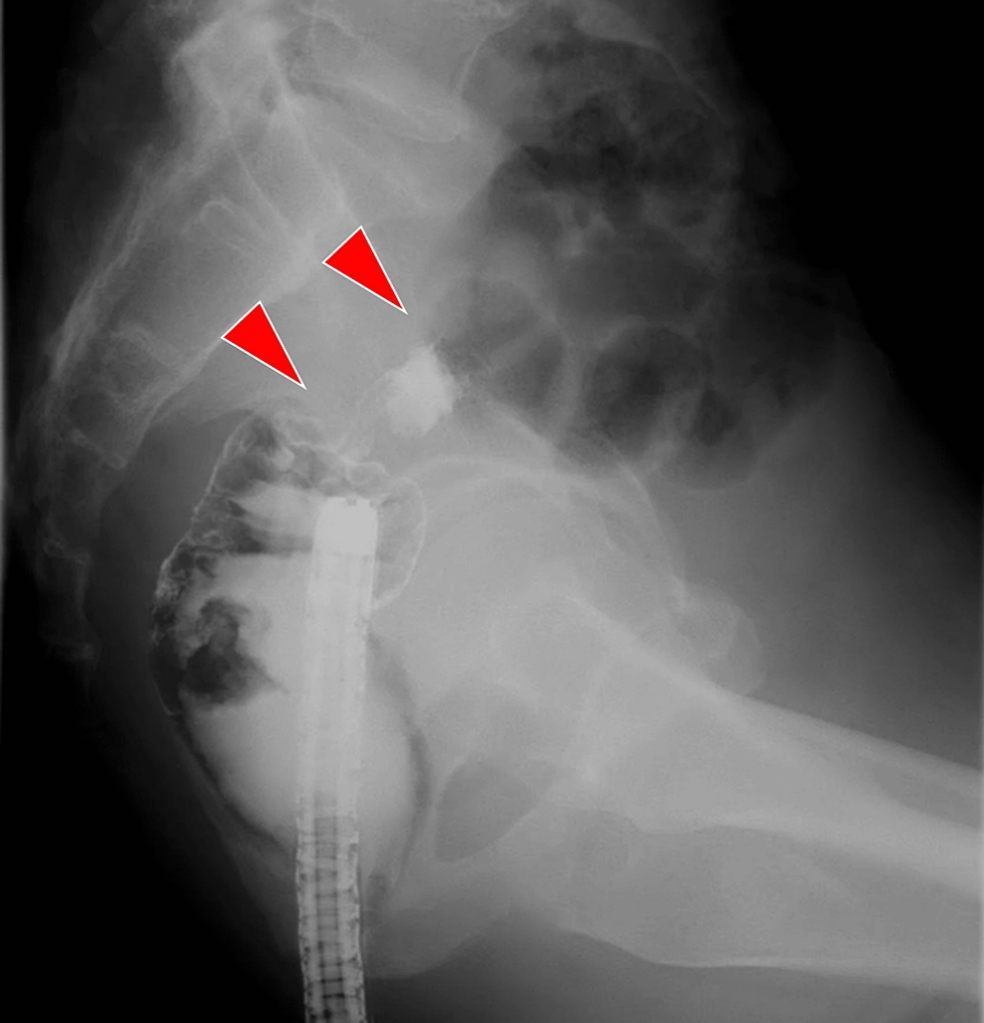
Fluoroscopic imaging with gastrografin contrast The obstructive lesion (arrowhead) in the upper rectum and the stenosis length were assessed via fluoroscopy using a contrast agent.

A 69-year-old man was referred to our hospital with complaints of anorexia, weight loss, and anorectal pain for three months; at this time, his CROSS score was 3. Since the lesion was located in the upper rectum and there were no obvious distant metastases, a stent was placed as a BTS. As previously mentioned, the widely used distal-opening stent is difficult to deploy with the distal side of the stent aligned precisely with the distal side of the lesion and can subsequently interfere with anorectal dissection. Therefore, we decided to use a proximal release-type colorectal stent. A 0.035-in guidewire and contrast catheter for endoscopic retrograde cholangiopancreatography (ERCP) were positioned at the distal side of the stenosis under endoscopic guidance, and fluoroscopic imaging with gastrografin contrast was used to confirm the site and extent of the stenosis (Figure 1).

**Figure 2:**
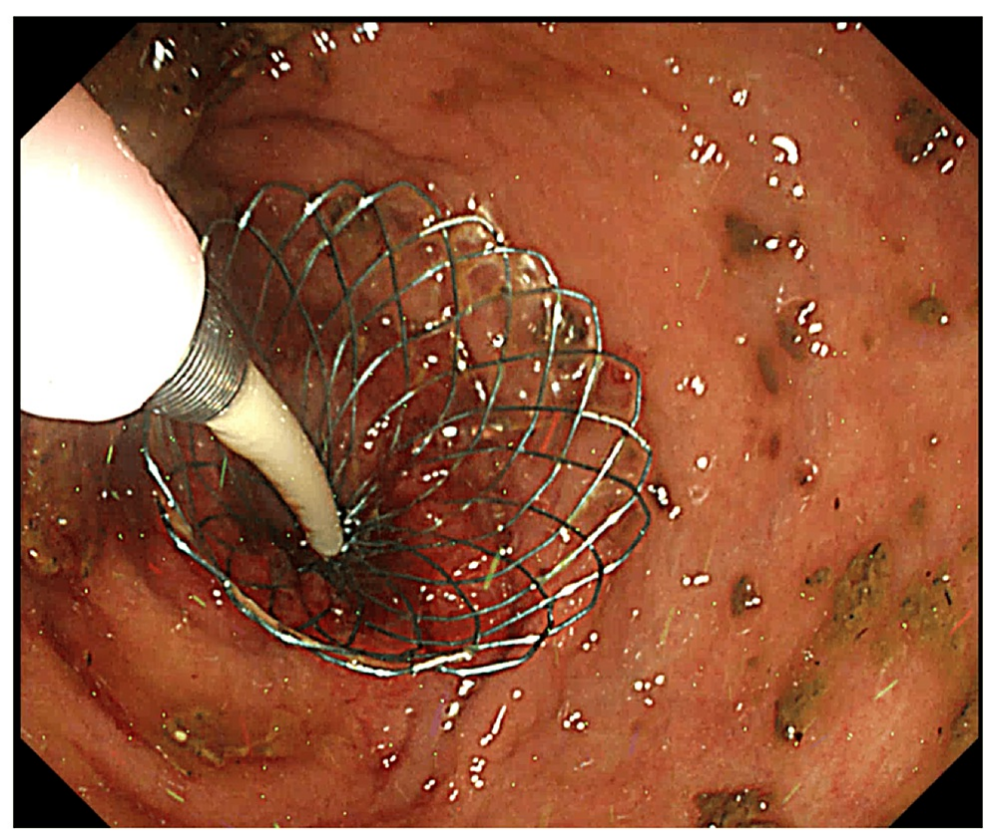
Colonoscopy image during stent deployment The scope was removed while leaving the guidewire across the stenosis and was reintroduced beside the guidewire. The delivery system was advanced over the wire, and the stent was released from the proximal side.

The endoscope and contrast catheter were removed once the guidewire was in place, after which the stent was inserted using the over-the-wire technique and released gently from the distal side, while appropriate positioning was maintained under fluoroscopic guidance. The distal edge of the stent was positioned at the distal edge of the tumor (Figure 2).

**Figure 3:**
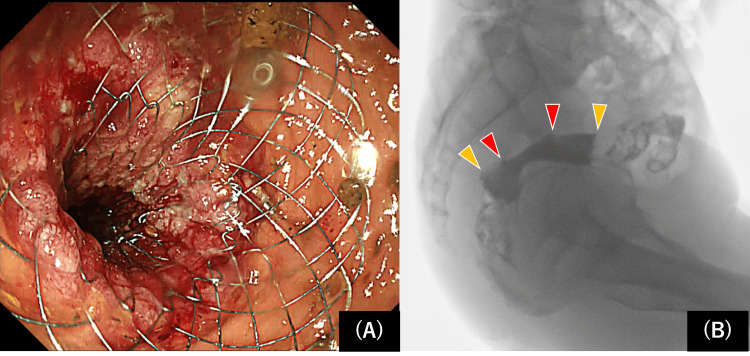
Colonoscopy and fluoroscopy images after stent deployment Stent deployment at a precise position under endoscopic (A) and fluoroscopic (B) guidance, indicating the edge of the tumor (red arrowheads) and stent (yellow arrowheads).

Conventional distal-release stents tend to be pulled toward the distal side of the lesion during deployment; therefore, it is necessary to deploy the stent while pulling it strongly toward the anorectal side of the lesion. However, the new proximal release-type stent was deployed by lightly pressing against the distal end of the lesion, making it easy to adjust the position and implant the stent so that it was aligned with the distal end of the lesion (Figure 3).

**Figure 4:**
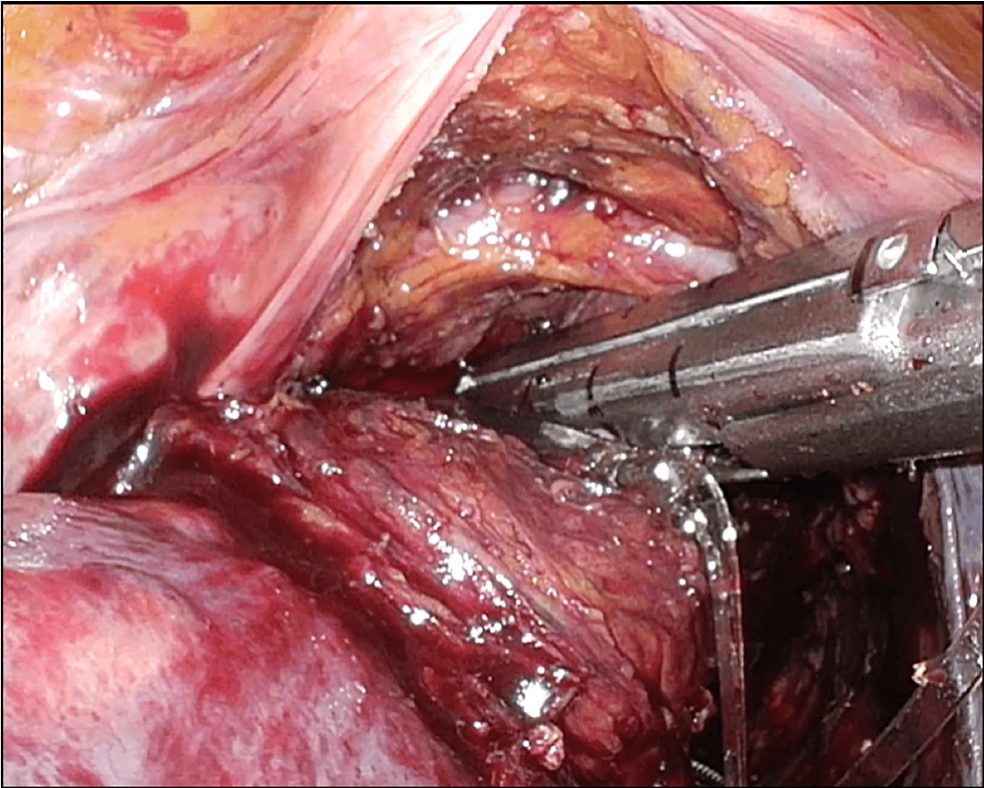
Intraoperative image of the anorectal dissection of the lesion The stent was placed without excess or deficiency on the anorectal side of the lesion, allowing anorectal dissection of the lesion without interference.

The time required for stent placement was 15 min, and the post-stent CROSS score was 4. The patient was allowed to resume a normal diet the day after stenting and was discharged from the hospital. Four weeks after stent placement, the patient was readmitted to the hospital and underwent low anterior resection. As the stent was placed in the correct position on the anorectal side of the lesion, anorectal dissection of the lesion was performed without any complications (Figure 4).

The operative time was 219 min, the patient lost 50 mL of blood, and no complications occurred during the procedure. The patient had a good postoperative course and was discharged seven days after the procedure without any adverse events.

## Discussion

Since January 2012, National Health Insurance in Japan has covered the use of SEMS for the treatment of malignant colorectal obstructions. This procedure is widely performed for preoperative decompression as a BTS and for palliative reasons [15-17].

Contraindications for SEMS placement include long or complicated stenosis, bleeding or inflammation, fistulas, and/or stenosis of the lower rectum near the anal verge. When evaluating the distance of the stent from the anal verge, clear contraindications are unknown due to individual differences; hence, there is a high likelihood of pain and distress when the stent margin approaches the dentate line of the anal canal. The documented rate of anorectal pain after conventional stent placement for lower rectal lesions was high (62.5%) [12]. Additionally, when placing a stent as a BTS, if the SEMS is placed closer to the anus than expected and causes pain, the surgical cutoff line will be lower than necessary. In rectal cancer surgery, the lower the anastomosis, the higher the incidence of suture failure, and the higher the risk of postoperative defecation dysfunction. Furthermore, the location of the anorectal end of the SEMS is important in cases of lower rectal stenosis because of the possibility of anastomotic complications, such as SEMS compression at the time of anorectal dissection. When using a conventional distal-release stent, the procedure and anatomy must be fully understood and the position of the SEMS must be carefully adjusted. However, because of the structure of the colon, conventional stents tend to be pulled toward the distal side during deployment, thus requiring a strong anorectal pull on the delivery system to maintain the proper implantation position. This makes it difficult to adjust the position of the anorectal side of the stent, and there is a high risk of the stent being located on the anorectal side, rather than in the correct position. The new proximal-release stent designed by Dr. Saida, a coauthor of this paper, became available in June 2019 and opened from the proximal side, allowing placement on the anorectal side of the lesion without excess or deficiency.

**Figure 5:**
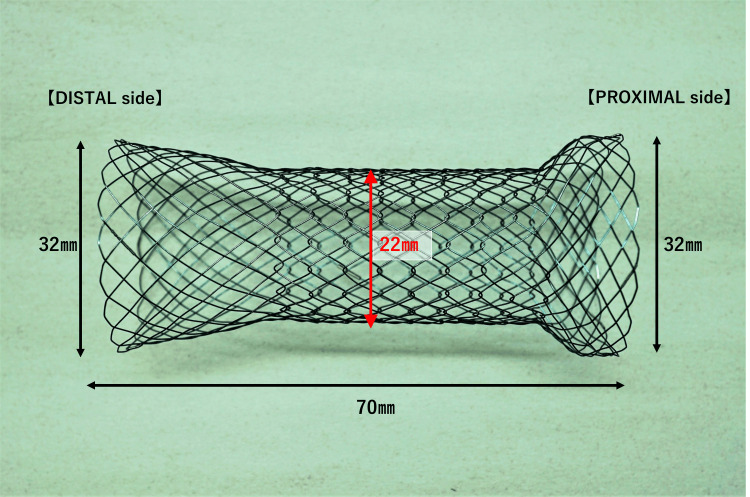
The newly developed proximal release-type colonic stent The newly developed proximal release-type colonic stent is 22 mm in diameter and 70 mm long and has flares (25 mm at the oral side and 15 mm at the anal side) at both ends to prevent the stent from straying.

The new proximal release-type stent (Niti-S Colonic Stent Proximal Release Type; Taewoong Medical Co., LTD, Gyeonggi-do, South Korea) used in the present study was 22 mm in diameter and 70 mm long with flares at both ends (25 mm on the oral side and 15 mm on the anal side) to prevent the stent from straying (Figure 5).

The stent had a closed-cell design and was foreshortened in 30% of patients. The delivery system was thick (16 Fr, 5.3 mm) and could not be placed through the scope; therefore, an over-the-wire technique was used.

In the present report, we experienced four cases and described one case of lower rectal cancer treated with stent placement as a BTS. None of the patients experienced complications associated with stent placement, and the CROSS score improved in all four patients. The distal margins of the resected specimens were 20-40 mm, indicating that the new proximal release-type stent allowed appropriate anorectal placement.

In addition to the BTS, the new proximal release-type stent may be useful for the palliative treatment of malignant stenosis of the lower rectum. In many cases, palliative treatment is associated with peritoneal dissemination and ascites. In some cases, it is difficult to construct a colostomy to remove any malignant colonic obstruction. Even if a colostomy is possible, the creation of a colostomy leads to a decline in the patient’s QOL. Additionally, once implanted, stents are difficult to remove, and the post-implantation anorectal pain and discomfort associated with stent placement can lead to a decline in QOL, especially in palliative cases. We are actively using this new proximal release-type stent for palliative purposes to treat malignant stenosis of the lower rectum.

## Conclusions

Conventional colonic stents tended to be placed closer to the anus than expected. This leads to pain, and the surgical cutoff line is lower than necessary when the stent is implanted in the rectum.

The new proximal release-type colonic stent aligned precisely with the anal verge and tumor under endoscopic observation and did not significantly affect the anorectal dissection line during low anterior resection, even after the stent was implanted. There were no post-placement complications in the cases described herein, including anorectal pain. The authors believe that this new type of stent will be useful in the future for the placement of malignant stenosis of the rectum.
